# Clinical Characteristics of Neuropathic Pain and Its Relationship with Cancer in Different Corporal Areas—A Systematic Review

**DOI:** 10.3390/diagnostics15010116

**Published:** 2025-01-06

**Authors:** Fernanda Danés-López, Cristóbal Diaz-Palominos, Anggie Ortiz Domínguez, Alanna Silva Rodriguez, Constanza Astorga, Daniela Martínez-Hernández, Juan Jose Valenzuela-Fuenzalida, Juan Sanchis-Gimeno, Pablo Nova-Baeza, Alejandra Suazo-Santibáñez, Gustavo Oyanedel-Amaro, Mathias Orellana-Donoso, Héctor Gutiérrez Espinoza

**Affiliations:** 1 Departamento de Morfología, Facultad de Medicina, Universidad Andrés Bello, Santiago 8370146, Chile; fdaneslopez@gmail.com (F.D.-L.); crismandipa@gmail.com (C.D.-P.); a.ortiz.domin@gmail.com (A.O.D.); daniela.martinezvh@gmail.com (D.M.-H.); juan.kine.2015@gmail.com (J.J.V.-F.); pablo.nova@usach.cl (P.N.-B.); 2 Faculty of Medicine and Science, Universidad San Sebastián, Santiago 8420524, Chile; alanna.elizabeth.lagos@hotmail.com (A.S.R.); constanzaastorga21@gmail.com (C.A.); mathor94@gmail.com (M.O.-D.); 3 Departamento de Ciencias Química y Biológicas, Facultad de Ciencias de la Salud, Universidad Bernardo O’Higgins, Santiago 8370993, Chile; 4 GIAVAL Research Group, Department of Anatomy and Human Embryology, Faculty of Medicine, University of Valencia, 46001 Valencia, Spain; juan.sanchis@uv.es; 5 Faculty of Health and Social Sciences, Universidad de Las Américas, Santiago 8370040, Chile; alej.suazo@gmail.com; 6 Facultad de Ciencias de la Salud, Universidad Autónoma de Chile, Santiago 8910060, Chile; g.oyanedelamaro@gmail.com; 7 Escuela de Medicina, Universidad Finis Terrae, Santiago 7501015, Chile; 8 One Health Research Group, Universidad de las Américas, Quito 170124, Ecuador

**Keywords:** cancer, neoplastic, tumor, neuropathic pain, therapy cancer, immunotherapy

## Abstract

**Background:** Neuropathic pain (NP) and cancer are caused by nerve damage due to cancer or treatments such as chemotherapy, radiotherapy, and surgery, with a prevalence that can reach up to 40%. Causes of neuropathic cancer pain (NCP) include direct nerve invasion or compression by the tumor, as well as neural toxicity associated with treatments. This type of pain is classified into several categories, such as plexopathy, radiculopathy, and peripheral neuropathies. **Methods:** Medline, Web of Science, Google Scholar, CINAHL, and LILACS databases were searched until October 2024. Two authors independently performed the search, study selection, and data extraction. Methodological quality was analyzed using the Robins-I tool. **Results:** The main findings of this review indicate that, depending on the cancer type, neuropathic pain will exhibit different characteristics, as well as identifying which types of cancer have a higher probability of presenting neuropathic pain. Additionally, there is a direct relationship whereby the more advanced the cancer, the greater the likelihood of experiencing neuropathic pain. Finally, although chemotherapy is employed as a cancer treatment, this therapy is quite invasive, and one of its adverse effects is that treated patients have a higher probability of developing neuropathic pain. **Conclusions:** Neuropathic pain is a condition that adversely affects patients with cancer. A detailed understanding of the relationships and triggers that produce this condition is present in only a small percentage of patients with cancer and is necessary to provide better treatment and gain a more comprehensive understanding of the characteristics of neuropathic pain. The objective of this study is to describe the relationship between different types of cancer or various treatments and the presence of NP.

## 1. Introduction

### 1.1. Neuropathic Pain in Cancer Patients

One of the most extensively studied clinical complications that individuals diagnosed with cancer may face is neuropathic pain (NP). This type of pain often emerges at various stages during the progression of the disease and is primarily a consequence of injury to the somatosensory system. Such injuries can arise from different sources, including trauma or lesions caused by tumors. The physiological mechanisms underlying NP have been linked to inflammation in the area surrounding the tumor, which is typically associated with the compression or infiltration of nearby tissues by the growing tumor itself. This process can lead to significant damage to the surrounding healthy tissues, exacerbating the pain experienced by the patient. Moreover, NP is not solely a result of the cancer itself; it has also been associated with various treatment modalities. These include surgical interventions, radiotherapy, and chemotherapy. In particular, NP is frequently referred to as chemotherapy-induced peripheral neuropathy (CIPN), highlighting the neurotoxic effects of chemotherapy agents. These effects are believed to disrupt axonal transport, ultimately leading to lesions in sensory neurons located in both the central nervous system and the peripheral nervous system [1,2].

### 1.2. Characteristics and Impact of Neuropathic Pain

Neuropathic pain is typically characterized as chronic and can be of high intensity, manifesting through a range of symptoms. Patients may experience numbness, hyperalgesia (increased sensitivity to pain), anxiety, allodynia (pain from stimuli that do not normally provoke pain), paresthesia (tingling sensations), depression, and sleep disorders. The presence of these symptoms can significantly disrupt daily activities, strain social relationships, affect economic situations, and ultimately diminish the overall quality of life for those who suffer from it. Interestingly, NP is reported to occur more frequently as a side effect of surgical treatments, particularly in patients undergoing procedures for breast cancer. Many of these patients describe their pain as sharp, urgent, or resembling an electric shock, predominantly localized in the thoracic region following surgery [3,4,5,6].

### 1.3. Challenges in Diagnosis and Management

**Figure 1:**
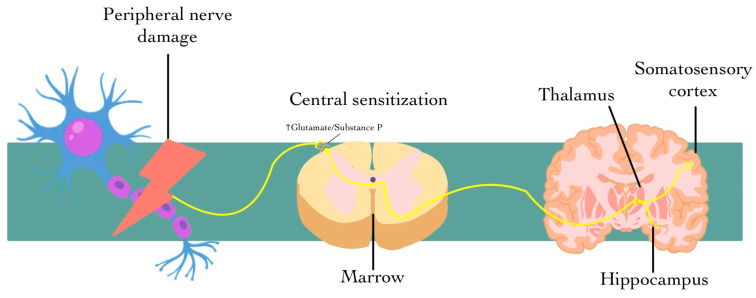
Damage to the peripheral nerve generates abnormal pain signals, while central sensitization in the spinal cord, driven by increased neurotransmitters like glutamate and substance P, amplifies these signals through neuronal hyperexcitability. These ascend to the thalamus, which relays them to the somatosensory cortex to process the location and intensity of the pain, and to the hippocampus, where the emotional component is integrated. This circuit perpetuates the physical and emotional pain characteristic of chronic neuropathic pain.

The challenge of diagnosing, characterizing, and effectively treating neuropathic pain represents a significant issue within the healthcare system. It is widely believed that early detection and monitoring of symptoms could play a crucial role in preventing the onset of NP. This belief underscores the urgent need for further investigation into the nuances of neuropathic pain, which could ultimately benefit patients who endure its debilitating effects. In conclusion, neuropathic pain is a complex and multifaceted condition that poses significant challenges for cancer patients. Understanding its mechanisms, symptoms, and impacts is essential for improving patient care and enhancing their quality of life [7,8,9] (Figure 1).

The objective of this study is to describe the relationship between various types of cancer or different types of treatments and the presence of NP.

## 2. Methods

### 2.1. Protocol and Registration

This systematic review and meta-analysis were performed and reported according to the Preferred Reporting Items for Systematic Reviews and Meta-Analyses (PRISMA) statement [10]. The registration number in the International Prospective Register of Systematic Reviews (PROSPERO) is CRD42022223061.

### 2.2. Eligibility Criteria

Studies that addressed the diagnosis of cancer and its association with the presence of neuropathic pain (NP) were included based on the following inclusion criteria: (1) Population: studies that reported patients diagnosed with cancer; (2) results: patients with various types and stages of cancer, including information on whether they had been exposed to chemotherapy treatments and whether, given this variety of scenarios, they presented a clinical diagnosis of NP in any body region; (3) studies: This systematic review included research articles, research reports, and original research published in English or Spanish in peer-reviewed journals indexed in some of the databases used to compile the studies. Conversely, the exclusion criteria were as follows: (1) Population: studies involving animals; (2) studies analyzing other types of chronic pain; (3) studies: letters to the editor or comments that did not provide data relevant to our research.

### 2.3. Electronic Search

We systematically searched MEDLINE (via PubMed), Web of Science, Google Scholar, the Cumulative Index to Nursing and Allied Health Literature (CINAHL), Scopus, and the Latin American and Caribbean Literature in Health Sciences (LILACS) from inception until January 2024. The search strategy included a combination of the following terms: “Cancer” (MeSH), “Neoplastic” (MeSH), “Tumor” (MeSH), “Neuropathic pain” (MeSH), “Cancer therapy” (non-MeSH), and “Immunotherapy” (non-MeSH), utilizing the Boolean connectors AND, OR, and NOT. For the PICO strategy, we extrapolated data to construct the following: Patients: Subjects with a diagnosis of cancer; Intervention: Treatment with immunotherapy; Comparison: Without immunotherapy; Outcomes: Presence of NP. The search strategies for each database are available in the Supplementary Material (Supplementary Table S1).

### 2.4. Study Selection

Two authors (FD and JV) independently screened the titles and abstracts of references retrieved from the searches. We obtained the full text for references that either author considered potentially relevant. A third reviewer (HG) was involved if consensus could not be reached.

### 2.5. Data Collection Process

Two authors (JV and MO) independently extracted data on the outcomes of each study. The following data were extracted from the original reports: (I) authors and year of publication, (II) type of study and number of participants, (III) incidence and characteristics, (IV) location of cancer, (V) geographic region, (VI) average age, (VII) sex, and (VIII) treatment.

### 2.6. Assessment of Methodological Quality

The methodology of each study, as well as the outcomes, was measured using the Risk of Bias in Non-Randomized Studies of Interventions (ROBINS-I) tool [11] during data extraction. ROBINS-I offers a comprehensive set of tools to assess and determine the risk of bias associated with confounding, participant selection, intervention measurements, deviations from the planned intervention, missing data, outcome measurements, and reported results. The ROBINS-I tool can be used in non-randomized cross-sectional and longitudinal studies, as quality is unaffected by the study design. Based on the literature on confounding, age, ethnicity/race, physical activity, and education were identified as the main confounders that needed to be appropriately adjusted for the study findings to be considered less risky. All areas are judged as being at low, moderate, severe, or critical risk of bias. Low risk indicates that a study is “similar to a successful randomized trial” in the area being evaluated. A moderate risk of bias means that the study is “good for a non-randomized study” but falls short of a full randomized trial. A very high risk of bias indicates that the study has “major problems”, while a very low risk of bias means the study is “too large...to provide useful information about the effects of the intervention”. If a domain does not contain enough information to determine whether it has a risk of bias, it is counted as having zero information. The total risk of bias for each article was the highest of all assessed fields [11].

## 3. Results

### 3.1. Selection of Articles

**Figure 2:**
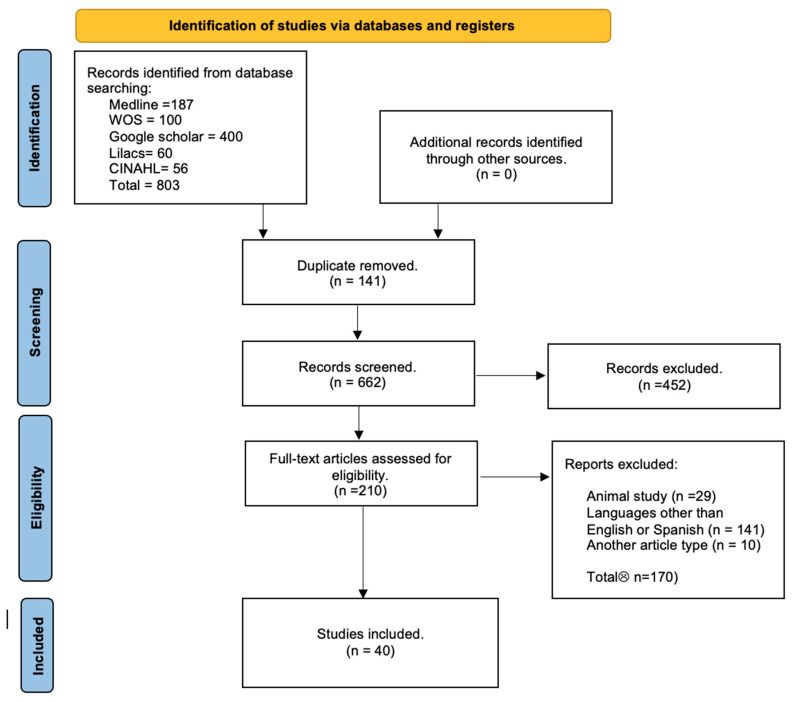
Search diagram for included studies.

The search process yielded a total of 803 articles from various databases, aligning with the criteria and search terms established by our research team. A filtration process was applied, focusing on the titles and/or abstracts of these articles. Out of the initial pool, 98 articles were included, and 40 articles were selected for inclusion in the systematic review. These articles were chosen based on their comprehensive study of the sample, detailed statistical data for each variant, and their utilization of a clear methodology (Figure 2).

A total of 40 articles were included in the final analysis. The total number of participants across all studies (N) was 7773. Of these participants, 2233 had NP associated with cancer or its treatments. The average age of the included participants was 52.49 [12,13,14,15,16,17,18,19,20,21,22,23,24,25,26,27,28,29,30,31,32,33,34,35,36,37,38,39,40,41,42,43,44,45,46,47,48,49].

### 3.2. Descriptive Results

Among the different types of research analyzed that address both the incidence and characteristics of NP in cancer patients, we observed that the prevalence of this type of pain varies according to the type of cancer and the treatment used. The types of cancer most frequently associated with NP are breast, lung, and colon cancer. Certain cancers with metastases in the bones, nervous system, or lymph nodes tend to present higher rates of NP (Table 1).

Oncological treatments that patients undergo, such as chemotherapy, are related to the incidence of NP. In studies such as those conducted by Cofeen and Garzón-Rodríguez [21,49], a high incidence of NP is observed in cancers like colon and breast cancer, where treatments with neurotoxic agents are common and typically involved in advanced stages of cancer (III and IV). Studies by Maña (2011) and Jiang [14,27] document an association between radiotherapy received by patients with cancers in the neck, oral cavity, and esophagus and the appearance of NP. On the other hand, the studies by Bokhari and Maña (2011) [14] show that post-surgical NP is common in patients with breast cancer.

Regarding interventions and treatments for NP, several pharmacological approaches are mentioned, such as opioids, anticonvulsants (like entin or pregabalin), tricyclic antidepressants (like amitriptyline), and topical treatments in the affected area with lidocaine. In studies such as that of Smith [38], mindfulness was used as a treatment for NP, moving away from more conventional therapies such as pharmacological ones. Casanova [50] also presents a type of non-pharmacological treatment, pretreatment, and pilot study where graded motor imagery (GMI) and neural mobilization (NM) techniques were applied. For his part, Lee used “Calmare” therapy as an intervention for NP in oncological patients, showing another alternative to the previously mentioned drugs, which may help reduce the dependence that patients can develop on these medications.

Several of the analyzed studies demonstrate statistically significant findings regarding the reduction of DN or the improvement of the quality of life of treated patients. In the study by Jiang [27], pregabalin exhibited a significant reduction in pain scores on the Numeric Rating Scale (NRS) compared to the placebo group. Mañas 2011 (B) [48] also indicates that pregabalin not only decreased the intensity of NP but also reduced the necessity for patients to use other medications, such as benzodiazepines.

Some research highlights the relationship between NP and factors such as sleep disorders, quality of life, and anxiety in patients with DN. Harada [52] shows that patients with DN experience a deterioration in quality of life compared to those without neuropathy. Fontes, on the other hand, demonstrated that the decline in sleep quality was greater in individuals with NP during the first year of follow-up. Juwara, meanwhile, found a significant association between DN and anxiety, indicating that patients with anxiety reported higher levels of pain, suggesting that emotional factors can exacerbate the pain in these patients.

Comparing the reviewed studies shows the diversity of therapeutic approaches used for the management of neuropathic pain (NP) in cancer patients. Acquazzino (2016) [16] observed that 26% of patients treated with chemotherapy, particularly those with leukemia and lymphoma, experienced NP, although a direct treatment for this pain was not specified in his cohort. In contrast, a more structured pharmacological approach was implemented in his Australian study with pediatric patients, using opioids and anticonvulsants, mainly gabapentin, for NP related to solid tumors such as osteosarcoma and Ewing sarcoma. On the other hand, Fallon (2015) [19] in the United Kingdom explored a topical and noninvasive alternative, noting that a 1% menthol cream was effective in reducing the intensity of NP in 82% of patients, including cases of colorectal cancer, breast cancer, and multiple myeloma. These results underscore the adaptability of NP management, where treatment varies based on the type of cancer, the clinical context, and individual patient needs.

The reviewed studies reflect a variety of cancer types and approaches to the treatment of NP, emphasizing its management according to the clinical context. Garzón-Rodríguez et al. (2013) [21] in Spain focused on patients with breast and lung cancer, where NP was linked to tumor progression and treatment, although no precise therapy was specified. Gordillo-Altamirano et al. (2016) [25] in Ecuador, without specifying treatment, highlighted the negative impact of NP on the quality of life in patients with breast cancer and lymphoma. Golan-Vered and Pud (2012) [23] in Israel observed chemotherapy-induced peripheral neuropathy in breast cancer patients treated with paclitaxel, although they also did not specify a treatment. Regarding the treatment of NP, Haumann et al. (2016) [24] in the Netherlands compared the use of methadone and fentanyl in patients with NP, finding that methadone provided faster pain relief, especially in unspecified cancers. Conversely, treatments for NP in cancer patients depend greatly on the type and stage of the disease. Jain et al. (2014) [26] in India analyzed 300 patients with various cancers and found that 10% had postoperative NP, although they did not mention a specific treatment. Jiang (2019) [27] in China evaluated pregabalin in 128 patients with head and neck cancers, showing that this anticonvulsant was effective in significantly reducing pain. In Canada, Juwara (2019) [28] studied 195 patients with postoperative breast cancer and found that anxiety increased the frequency of NP without reporting a direct treatment. Lee et al. (2016) [31] in South Korea tested Calmare Therapy in 26 patients with various types of cancer, noting that this noninvasive technique reduced post-surgical and chemotherapy-induced NP. In Spain, López-Ramirez et al. (2016) [32] evaluated 5% lidocaine patches in 15 patients with different cancers, achieving effective relief of NP in 79.9% of cases. Collectively, these studies emphasize how approaches to treating NP vary based on the type of cancer and patient-specific factors.

**Figure 3:**
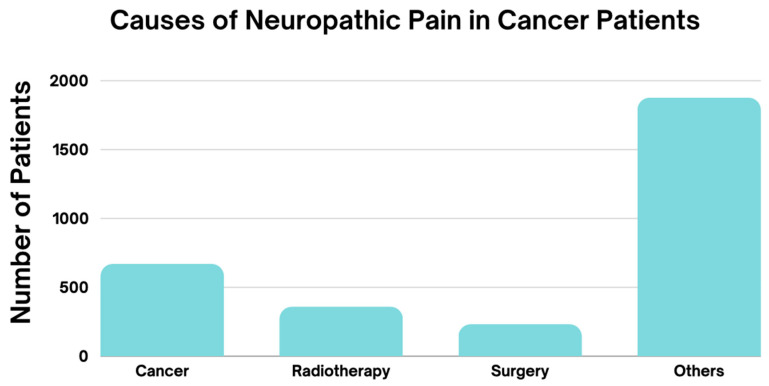
The graph illustrates the primary causes of neuropathic pain in cancer patients. The most frequent cause is “Other”, which includes chemotherapy, medications that affect the nervous system such as pregabalin, non-narcotic analgesics, narcotic analgesics, benzodiazepines, antidepressants, and anticonvulsants, as well as lidocaine and Calmare therapy. In addition to these treatments, pain is also assessed using various pain scales and scoring systems, affecting 1877 patients. “Cancer” itself follows as the second most common cause, with 670 patients, while neuropathic pain associated with radiotherapy and surgery is reported in 359 and 232 cases, respectively. These findings underscore the significant role that oncological treatments, along with pain assessment tools, play in the development of neuropathic pain, highlighting the need for specialized strategies to manage this condition effectively.

**Figure 4:**
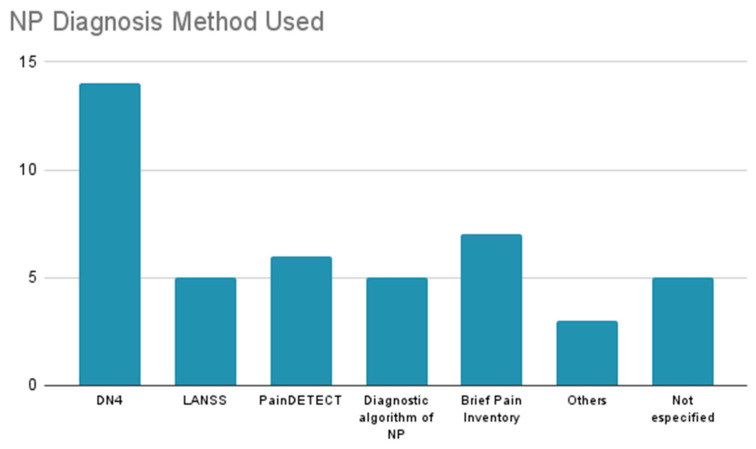
This graph shows the number of articles in which each method was utilized for the diagnosis of neuropathic pain. The most frequently used method was the DN4 Questionnaire, which appeared in 14 articles.

**Figure 5:**
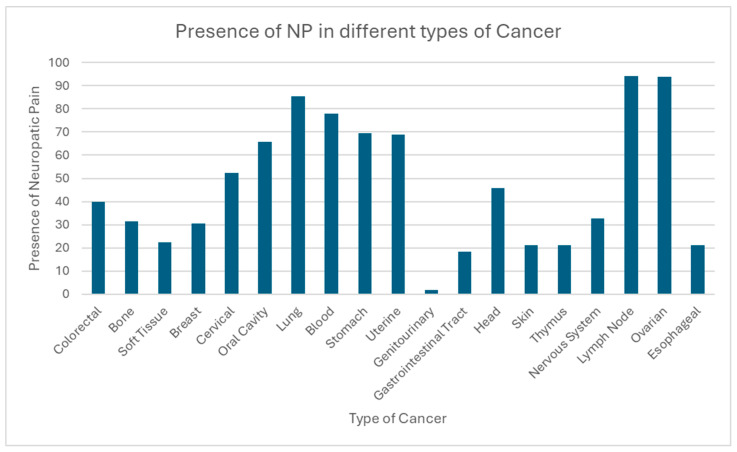
This graph shows the presence of neuropathic pain in different types of cancer.

The prevalence of NP in cancer patients varies considerably depending on the type of cancer, the treatment applied, and the individual characteristics of each patient. Cancers such as breast, lung, and colon, along with those with bone, nervous system, or lymph node metastases, show a higher incidence of NP, particularly in advanced stages. Cancer treatment, especially chemotherapy and radiotherapy, is closely associated with the occurrence of this type of pain, particularly when neurotoxic agents are used. Additionally, various therapeutic approaches, both pharmacological and non-pharmacological, have been identified for the management of NP, including opioids, anticonvulsants, tricyclic antidepressants, topical lidocaine, and innovative therapies such as mindfulness and Calmare therapy. The efficacy of these treatments varies depending on the clinical context and the type of cancer, making it crucial to personalize the therapy according to the individual needs of the patient. Furthermore, emotional factors such as anxiety and sleep disorders also play a significant role in the intensification of pain, highlighting the importance of a comprehensive approach in the management of NP. The findings underline the need for tailored treatment that considers not only the type of cancer but also the physical and emotional well-being of patients (Figure 3, Figure 4 and Figure 5, Table 2).

#### 3.2.1. Large Intestine Cancer

Among the types of cancer that we have been able to study in relation to NP, there is cancer that affects the large intestine. Nine articles were reviewed, all of which involved patients presenting NP; however, only five patients received treatment for this NP. In seven of the studies, the patients underwent chemotherapy, while in two studies, it is unspecified whether the patients received any type of oncological treatment. Additionally, it was noted that the stages of cancer varied according to the study: in one article, all stages were considered, while in four studies, patients in more advanced stages (III to IV) predominated.

#### 3.2.2. Bone Cancer

Regarding bone cancer, six articles were reviewed, all of which involved patients presenting NP; however, only three reported a treatment for this pain. In four of the studies, the patients underwent chemotherapy or radiotherapy, while one study did not specify whether the patients received any oncological treatment. Furthermore, it was observed that the cancer stages of the involved patients varied according to the study. In three of the articles, all stages were considered, while two articles did not specify the cancer stage of their patients.

#### 3.2.3. Soft Tissue Cancer

In relation to cancer affecting soft tissues, five studies were reviewed, all of which involved patients presenting NP; however, only two reported a treatment for this pain. In two of the studies, the patients had undergone chemotherapy or radiotherapy or were post-surgical patients, while in one study, only post-surgical patients were considered, and in another article, it was not specified whether the individuals underwent any treatment for cancer. Likewise, in three of the articles, the stages of the patients’ cancer were not specified. In one of the studies, all stages were considered, while in another, there was a predominance of patients in more advanced stages of cancer (III to IV).

#### 3.2.4. Genital Cancer

Regarding cancer affecting the female genital organs, three studies were reviewed, all of which included subjects with NP; however, no article proposed a treatment for this pain. In one of the studies, patients had undergone chemotherapy or radiotherapy or were post-surgical patients, while in another study, no type of oncological treatment was specified, and in a third study, only patients who had undergone chemotherapy were considered. On the other hand, in two of the investigations, patients at all stages of cancer were considered, while one article focused specifically on patients with metastasis (stage IV).

#### 3.2.5. Breast Cancer

**Figure 6:**
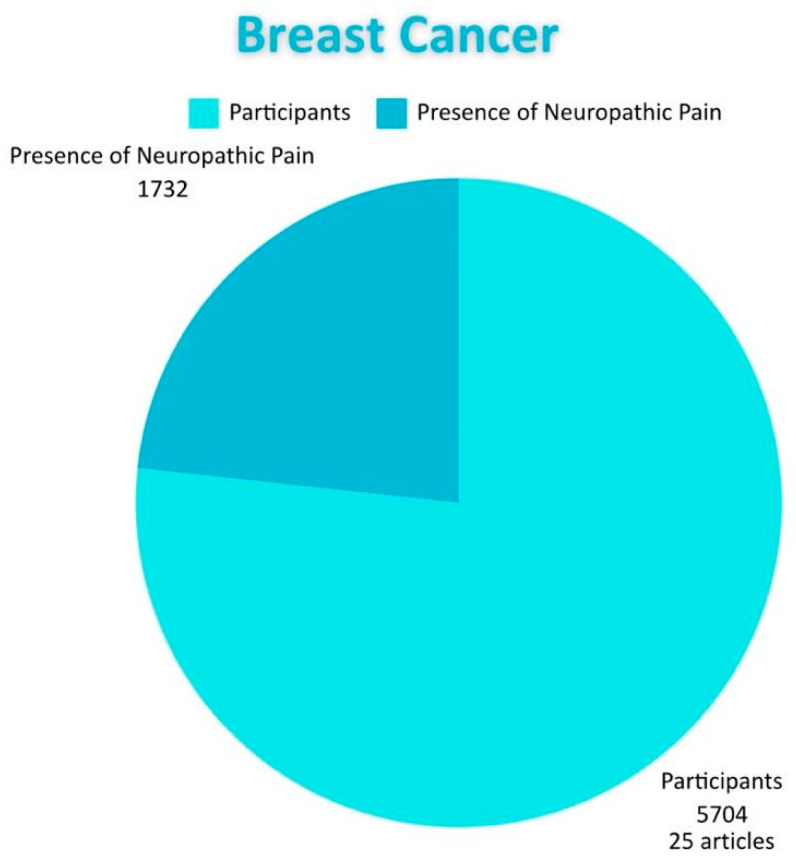
Shows the distribution of subjects with breast cancer and the proportion of how many of them have neuropathic pain.

Among all the articles reviewed, breast cancer is the most common type of cancer, being addressed by 25 of the studies. All patients in these studies had NP, but only 10 studies reported treatment for this pain. In six of the studies, patients had undergone chemotherapy or radiotherapy or were post-surgical patients. In five of the studies, only patients who received chemotherapy were considered, while in two articles, only patients who underwent radiotherapy were included, and in three studies, only post-surgical patients were examined. In three studies, patients had undergone only chemotherapy or radiotherapy, while in another five studies, it was not specified whether the subjects received any type of cancer treatment. Furthermore, it was observed that in 10 studies, the stage of cancer was not specified. In 10 articles, patients at all stages of this disease were considered, while in five studies, the focus was primarily on more advanced stages of cancer (stage IV) (Figure 6).

#### 3.2.6. Cervical Region Cancer

In relation to cancer affecting the neck, seven articles were reviewed, in which all subjects had NP; however, in only four of the studies was a treatment for this pain proposed. In two of the publications, the patients had been treated with chemotherapy or radiotherapy. In one of the studies, the patients were subjected only to chemotherapy, while in two articles, the individuals had received only radiotherapy, and in one study, they were only post-surgical patients. In one of the articles, it is not specified whether the subjects involved had undergone any type of oncological treatment. Similarly, in three of the studies, the stage of the patients’ cancer is not specified, while in two studies, individuals from all stages participated, and in two, they focused exclusively on patients with metastasis (stage IV).

#### 3.2.7. Oral Cavity Cancer

Addressing the types of cancer affecting the oral cavity, four studies were reviewed, in which all patients presented NP, and in all the articles, some type of treatment for this pain was proposed. In one of the publications, individuals who had undergone chemotherapy or radiotherapy were considered, while in two articles, only individuals who received radiotherapy were included; one of the studies specified whether the people involved received any type of treatment for cancer. Regarding the stages of the patient’s cancer, in two articles, all stages were considered, while in one, they focused only on patients in stages III to IV, and in another publication, they did not specify the stage of cancer for the subjects involved.

#### 3.2.8. Lung Cancer

**Figure 7:**
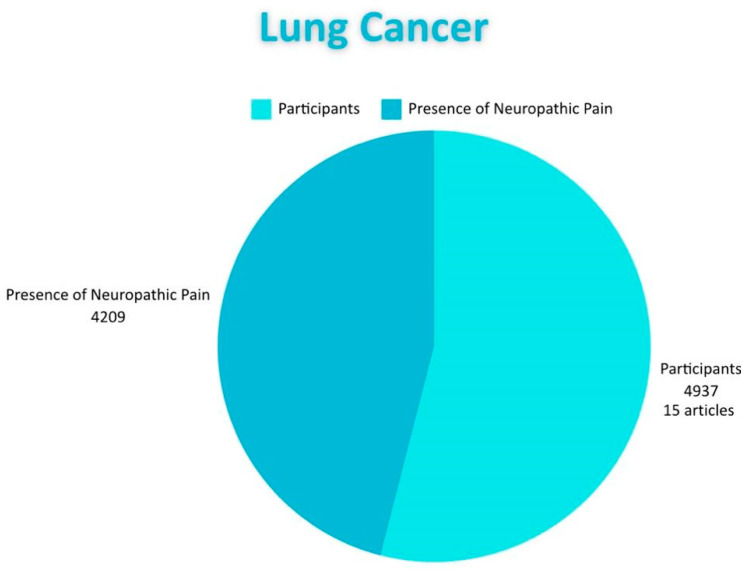
Shows the distribution of subjects with lung cancer and the proportion of how many of them have neuropathic pain.

Regarding lung cancer, 15 articles related to NP were reviewed. Only eight of them presented a treatment to reduce pain. In 10 of the publications, patients underwent chemotherapy; nine articles indicated that patients received radiotherapy, and four articles involved post-surgical patients and patients but did not specify patients with metastasis. Furthermore, cancer was also observed. One of the articles considered only patients with stages I to III; five studies addressed patients with all stages, i.e., I–IV; another five studies focused solely on patients with stage IV; two studies included only patients with stages III–IV; and only three studies did not specify (Figure 4 and Figure 7).

#### 3.2.9. Liver Cancer

In relation to liver cancer, four articles associated with NP were studied, and only one indicated a treatment to reduce pain. In all the articles studied, the patients were subjected to chemotherapy, two to radiotherapy, and two were post-surgical. On the other hand, the different stages of the cancers were also observed: three of them in stage IV and one article with patients in stages III–IV.

#### 3.2.10. Hematologic Cancer

**Figure 8:**
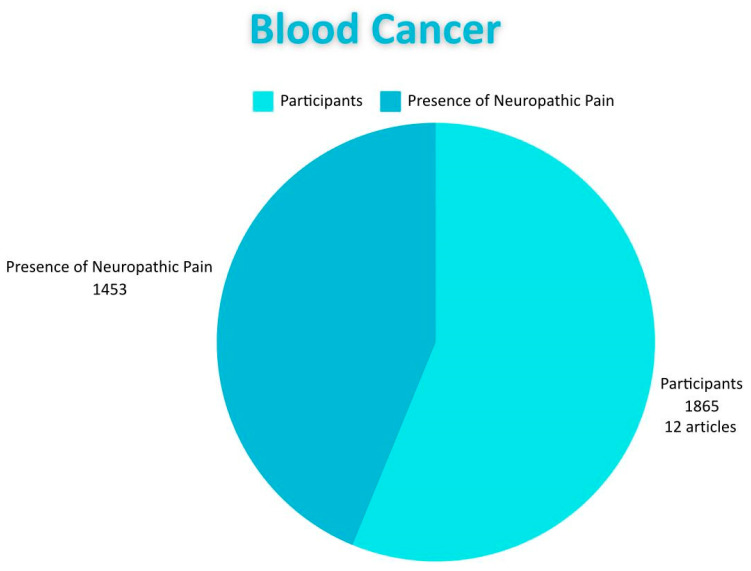
Shows the distribution of subjects with lung cancer and the proportion of those who have neuropathic pain.

Regarding blood cancer, 12 articles were studied, all of which were related to NP, but only eight of them presented any treatment for this pain. Ten articles involved subjects undergoing chemotherapy, another six articles involved subjects undergoing radiotherapy, four articles reported subjects who were post-surgical, one article discussed patients with metastases, and two articles did not specify. Regarding the stage of cancer, three articles indicated that the subjects were in all stages, that is, from I–IV. Another two indicated only stage IV, four articles did not specify, one was in stage III, and finally, two articles were in stages III–IV (Figure 5 and Figure 8).

#### 3.2.11. Stomach Cancer

Regarding stomach cancer, four articles were studied, all of which were related to neuropathic pain, but only one presented a treatment to reduce pain. In three of the articles studied, it was stated that the patients underwent chemotherapy, two underwent radiotherapy, two were post-surgical, and only one did not specify. Speaking about the stage of cancer, two had patients in stage IV. Another article indicated that the patients had cancer at all stages (I–IV), and one article considered patients in stages III to IV.

#### 3.2.12. Pancreatic Cancer

Regarding pancreatic cancer, three articles were collected, all of which reported patients presenting with neuropathic pain, but none mentioned treatment for this pain. In three of the articles, patients were presented as undergoing chemotherapy, two underwent radiotherapy, and two articles indicated that the patients were post-surgical. Regarding the stage of cancer, all three indicated that patients were in the terminal stage (IV).

#### 3.2.13. Bile Duct Cancer

Within bile duct cancer, two articles were studied that were related to neuropathic pain, and neither discussed any treatment for this pain. In both studies, it was indicated that the patients underwent chemotherapy, but only one mentioned that the patients also received radiotherapy and were post-surgical. Regarding the stage of cancer, both articles indicated that the patients were in the terminal stage (IV).

#### 3.2.14. Uterine Cancer

Regarding uterine cancer, four studies were collected, all of which dealt with neuropathic pain, and two of them presented any treatment for it. All articles indicated that the patients underwent chemotherapy; two had radiotherapy, three involved post-surgical patients, and one had metastasis. Speaking about the stages of cancer, three articles indicated that the patients were in stage IV and indicated that the patients were in stages III–IV.

#### 3.2.15. Urinary Tract Cancer

Regarding genitourinary cancer, four studies were compiled, all of which were related to neuropathic pain, but three of them presented any treatment for this pain. Two articles indicated that the patients underwent radiotherapy, another article presented only post-surgical patients, one article involved patients undergoing chemotherapy chemotherapy, and finally, one did not specify. Regarding the stages of cancer, two articles indicated that the patients were in all stages, i.e., I–IV, and two did not specify.

#### 3.2.16. Bowel Cancer

In relation to gastrointestinal cancer, five studies were examined, and all of them related to neuropathic pain, but only three of them presented any treatment for this pain. Concerning what the patients were subjected to, two articles indicated that the patients underwent radiotherapy, another two articles indicated that the subjects underwent chemotherapy, one article did not specify, and one specified post-surgical patients. Regarding the stages of cancer, three articles did not specify, but two of them indicated that the patients were in all stages, namely from I–IV.

#### 3.2.17. Head Cancer

Within head cancer, six studies were compiled, all of which were related to NP; however, only three of them presented any treatment for this pain. Three articles indicated that the patients underwent radiotherapy, another three indicated that the patients underwent chemotherapy, another article presented post-surgical patients, and one did not specify. In relation to the stage of cancer, of the total studied, two articles indicated that the patients were in all stages (I–IV), another two were in only stage IV, and finally, another two were not specified.

#### 3.2.18. Skin Cancer

Regarding skin cancer, two articles were studied, both of which were related to NP and also presented some treatment for this pain. One of them indicates that the patients underwent chemotherapy, and both of the patients underwent radiotherapy. Regarding the stage of cancer, both studies present patients who were in all stages, i.e., I–IV.

#### 3.2.19. Thymus Cancer

In relation to thymus cancer, two articles were studied, and both were restudied. The NP also presented some treatments for this pain. One of them indicates that the patients underwent chemotherapy, and both patients underwent radiotherapy. In relation to the stage of cancer, both studies present patients who were in all stages, that is, from I–IV.

#### 3.2.20. Nervous System Cancer

Within the field of nervous system cancer, three articles were studied, all of which were related to NP; however, only two of them presented any treatment for this pain. In two articles, the patients were subjected to chemotherapy; in all three articles, the patients were subjected to radiotherapy; and in only one article were there post-surgical patients. In relation to the stages of cancer, two articles indicate that the patients were in all stages, i.e., I–IV, and only one did not specify.

#### 3.2.21. Lymphatic Cancer

Regarding lymph node cancer, five articles were studied, all of which were related to NP, but only three presented a treatment for it. Four of the articles studied indicate that their patients underwent chemotherapy, three other articles indicate that their patients underwent radiotherapy, two articles indicate that they included post-surgical patients and one of them did not specify. Regarding the duration of cancer, two articles indicated that the patients were in all stages, i.e., I–IV; one article indicated that their patients were in stages III and IV. Another article indicates only stage IV, and finally, the last article did not specify.

#### 3.2.22. Ovarian Cancer

In relation to ovarian cancer, two articles related to NP were studied, both of which presented some treatment for this pain. Both articles indicate that their patients underwent chemotherapy, but only one of them states that their patients underwent radiotherapy. Regarding the stage of cancer, both articles indicate that their patients were in stages III and IV.

#### 3.2.23. Esophageal Cancer

Within esophageal cancer, two studies were examined, both of which were related to neuropathic pain (NP), and both presented some treatment for this pain. Both articles indicate that the patients underwent chemotherapy, but only one article indicates that, in addition, their patients underwent surgery, with some having metastasis. Regarding the stages of cancer, one article indicates that it only involved patients with stage IV, while the other article mentions subjects in stages III and IV.

#### 3.2.24. Prostate Cancer

Regarding prostate cancer, three studies were examined, only one of which had a treatment for NP. Two of the three articles involved patients who had undergone chemotherapy; two other articles indicate that their patients received radiotherapy, and one of the articles mentions post-surgical subjects. Concerning the stage of cancer, two articles indicate that the subjects were in the terminal stage, i.e., stage IV, while one of them does not specify.

#### 3.2.25. Laryngeal Cancer

In relation to laryngeal cancer, two articles were examined regarding NP, and both presented a treatment for this pain. In both articles, the patients underwent radiotherapy, but only in one were there post-surgical subjects. Regarding the stage of this cancer, neither article specifies the stage.

#### 3.2.26. Pharyngeal Cancer

In relation to pharyngeal cancer, three articles related to NP were examined; however, only one of them presented any treatment for this pain. In all three articles, the patients underwent radiotherapy, but only one article indicates that the patients also received chemotherapy, and some subjects were post-surgical. Concerning the stage of cancer, only one article indicates that the patients were in stage IV, while the other two articles do not specify.

### 3.3. Risk of Bias of Included Articles

**Figure 9:**
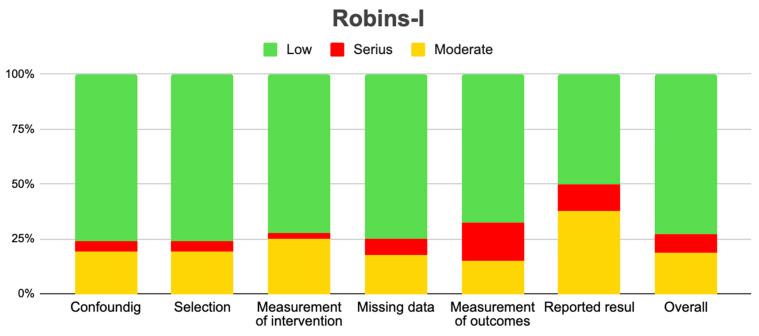
Bias diagram of the included articles according to the Robins-I analysis tool.

The summary of the risk of bias in the included studies using the ROBINS-I tool is shown in Figure 6. Of the included studies, four had a high risk of bias [12,19,32,40], while two had some concerns [13,17]. Those with serious risks encountered significant problems with the selection of study participants and also exhibited moderate bias in the classification of interventions, so these studies should be treated and analyzed with greater caution based on the interpretation of their results (Figure 9).

## 4. Discussion

NP has neurophysiological principles that can be associated with a significant number of pathologies, which may accentuate the clinical symptoms of these patients. One of the conditions directly linked to the presence of NP is cancer. We have demonstrated that depending on the cancer type, NP may be observed to have a greater prevalence in certain cancers. Moreover, there is a higher prevalence of NP in patients who have undergone chemotherapy, with symptoms increasing or exacerbating in specific cancers that present NP due to chemotherapy.

Breast cancer stands out among the reviewed types of cancer as the one most frequently associated with NP. The management of NP is a crucial aspect, given its impact on the quality of life of patients. This pain can affect individuals both physically and emotionally, intensifying the suffering of those facing a serious prognosis. We found that 25 articles address breast cancer, involving a total of 5704 patients, making it the most prevalent cancer compared to the others included in the study. A relevant aspect of this type of cancer is its frequent association with advanced stages of the disease, especially stage IV, where treatment is more oriented toward palliative care than a curative intervention. The number of patients diagnosed with NP or experiencing recurrent symptoms increases. This lack of specific treatment for NP is reflected in our review, as only 10 of the 25 studies reviewed regarding breast cancer present a treatment for NP. Regarding the association of this type of pain with different interventions performed in cancers, we found 11 studies, including 703 patients who presented NP after surgery. Conversely, in a study that involved 359 patients, this type of pain associated with chemotherapy was documented. Furthermore, in a total of 670 patients, NP was observed as directly related to cancer. All of the above highlights the importance of addressing this type of pain, given its significant impact on the quality of life of patients. Another of the most frequent cancers associated with NP is lung cancer. Fifteen studies were reviewed, with a total of 4937 participants. An important characteristic of this pain is the high incidence rate of NP associated with cancer itself; 10 studies with 2100 participants detail this association. It is essential to know how many studies propose a treatment for pain related to this cancer; only eight indicated one. Likewise, NP associated with post-surgeries (related to cancer) was identified in six studies involving this parameter and 232 participants. Finally, NP associated with chemotherapy was documented in nine studies with 1877 participants. Lung cancer is particularly significant due to various predisposing factors, such as tobacco consumption, environmental smoke, electronic cigarettes, and cigarettes. Speaking of other cancers related to NP, we found blood cancer in 12 articles involving a total of 1865 participants, with only eight presenting a possible treatment for this pain. Eight studies specifically related to neuropathic pain due to blood cancer encompassing 402 patients with this pain. Conversely, only three studies involving a total of 52 patients address neuropathic pain post-surgery. Finally, there are four studies encompassing a total of 999 participants. It is vital to consider this cancer since it affects the production of erythrocytes and red blood cells and also impacts their function. Among various types are acute lymphocytic (or lymphoblastic) leukemia (ALL), acute myeloid leukemia (AML), chronic lymphocytic leukemia (CLL), and chronic myeloicemia (CML) in addition to lymphoma (CML), myelomas, and others. Preventing the development of these cancers is essential as they progress quickly and can affect multiple body systems, weakening the immune system and leading to complications such as infections and bleeding. Furthermore, treatment can be expensive and carry high risk, negatively impacting the quality of life and emotional well-being of patients. Early detection and prevention, when possible, can significantly improve prognosis. Colorectal cancer also shows a notable association with NP, as evidenced by the nine reviewed articles that included a total of 1666 patients. Among these, five studies with 598 patients reported NP associated with chemotherapy treatments, highlighting the need to address this effect to improve patients’ lives. Additionally, three studies described the presence of NP after surgical interventions. Furthermore, two studies with 33 patients documented NP as directly associated with colorectal cancer. Regarding the treatment of NP, five of the studies proposed a treatment approach for this pain. In terms of the stages considered in these nine publications, five focused mainly on later stages (III–IV), while the remaining four (III–IV) did not specify the patients‘ stages.

Finally, in relation to the cancer with the highest prevalence of NP, those that presented the most NP were mammary gland, lung, colorectal, and blood cancers. Although a physiological relationship has not been established to explain why these regions with pathology could have a higher prevalence of NP, this could be associated with the fact that in the early stages, these cancers are often more silent, which is expressed through minimal evident symptoms. In higher stages, such as type IV, where metastasis has already occurred, it becomes more aggressive and may generate neuroplastic tissue changes that could be attributed to predisposing factors for NP. On the other hand, concerning cancer treatment, the highest predisposition to NP is observed after surgery for soft tissue cancer (*p* = 0.001). After chemotherapy, there was a higher probability of NP in blood cancer, gastrointestinal cancer, and pharyngeal cancer. Finally, only two studies showed a greater difference regarding post-surgical and post-chemotherapy pain, specifically pharyngeal cancer and oral cavity cancer. All these results indicate that NP is present in several patients with cancer, which can be multifactorial and not due to a clear or defined onset (Table 3).

Regarding previous review studies that have examined NP and its association with cancer, we identified two studies that associate NP with cancer. The article by Mulvey [54,55] revealed that various types of pharmacological and intervention treatments aimed at addressing NP are effective in patients with NP. The methodology of this review included 93 studies, of which only three are present in our study. This article reports that there is a lack of standardized guidelines for managing NP, constituting a barrier to effective treatment and diagnosis. While it establishes a relationship between NP and cancer, it focuses on treatment, whereas our review is centered on the characterization of the relationship between these two conditions and what should also be taken into consideration for managing patients with cancer. The study by Roberto et al. evaluated the prevalence of NP, concluding that 1 in 3 patients with cancer also report NP. This article examined 40 studies, three of which were included in our review. Conversely, our work encompasses 28 more recent articles than the publication of this review. The relationship we address in this study is treated very superficially and is based on only two studies, which is why our study differs significantly from the one mentioned above. Finally, in the article by Yon et al., the prevalence of NP and its effect on the quality of life are evaluated, with an emphasis on the pathophysiology and management of patients with NP and cancer. In conclusion, it is reported that the prevalence of NP reaches 40% of cancer patients, while in our study, it does not exceed 30% of patients with NP and cancer. It is also noted that this pain significantly impacts the quality of life of affected individuals, even more so than nociceptive pain. Furthermore, management is described as complex, including anticonvulsants, antidepressants, combined opioid therapy, topical agents, and intervention strategies. In relation to these previous studies, our review has a distinct and less-studied objective.

## 5. Limitations

This review was limited by the publication and authorship biases of the included studies. First, studies with differing results that were in non-indexed literature in the selected databases may have been excluded. Second, there could be limitations in the sensitivity and specificity of the searches. Finally, the authors personally selected articles. All of this increases the likelihood of excluding potential cases from countries outside of Asia and North America that are not reported in the scientific community.

## 6. Conclusions

The coexistence of cancer and neuropathic pain (NP) is evidenced in the literature. This duality of simultaneous conditions significantly alters patients’ quality of life. We believe that knowing the region affected by cancer has the highest probability of producing NP is crucial. In relation to this, when these types of cancer are diagnosed, the treating physician should suggest some form of preventive therapy to help reduce the risk of developing NP. A concerning factor is that certain therapies may increase the probability of NP. More studies are needed to address the pathophysiological events that mediate the presence of NP associated with these therapies, and its association should prompt new studies to enhance understanding of the condition, thus enabling the development of new guidelines for the prevention and effective treatment of NP associated with cancer.

## Figures and Tables

**Table 1:** Characteristics of the included studies.

Author, Year	Type of Study and Number of Participants	Incidence and Characteristics	Location of Cancer	Statistical Values and Characteristics	Geographic Region	Average Age	Sex	Treatment
Acquazzino et al., 2016 [16]	Prospective cohort study: 78 patients	Leukemia 32 (41%), solid tumor 20 (26%), lymphoma 18 (23%), CNS tumor 8 (10%). 75 (95%) patients underwent chemotherapy.	Red blood cells, white blood cells, and central nervous system.	There were no statistically significant differences in the sex of the patients (*p* = 0.06). There was no statistically significant difference between solid tumors and leukemia (*p* = 0.36). There was no statistically significant difference between lymphoma and leukemia (*p* = 0.17). There was no statistically significant difference between CNS tumors and leukemia (*p* = 0.07). There was no statistically significant difference between patients who received radiotherapy and those who did not receive radiotherapy (*p* = 0.08).	USA	40.4	41 (53%) males, 37 (47%) females	No treatment is mentioned. Quantify patients receiving chemotherapy who have neuropathic pain (26%).
Anghelescu et al., 2015 [15]	Retrospective study of 323 patients	Fifty-six (17%) of these patients had PN (34 males and 22 females), and 66 referrals (15%) were for PN. Solid tumor (37 of 56, 66.1%); solid tumors were osteosarcoma (24 patients (42.9%)), Ewing’s sarcoma (8 (14.3%)), Acute lymphoblastic leukemia (6 (10.7%)), Acute myeloblastic leukemia (4 (7.1%)), Non-Hodgkin’s lymphoma (4 (7.1%)), brain tumor (4 (7.1%)), sarcoma (2 (3.6%)), synovial sacroma (1), osteochondroma (1 (1.8%)), Rhabdomyosarcoma (1 (1.8%)), Hodgkin’s lymphoma (1 (1.8%)).	Bones, red blood cells, white blood cells, lymph nodes above the diaphragm, brain, and soft tissues.	The NP patient group had 1401 clinic visits (778 inpatient visits [55.5%] and 623 outpatient visits [44.5%]). NP patients had a significantly higher mean number of pain visits per consultation (*p* = 0.008) and significantly more days (median) of pain service follow-up (*p* < 0.001) than other patients. The most common cause of PN was cancer treatment rather than underlying malignancy. Pharmacologic management of PN was complex, often comprising 3 medications. Nonpharmacologic approaches were used for 57.6% of NP referrals.	Australia	45.9	34 (60.71%) men with PN. 22 (39.29%) women with PN.	Opioids, anticonvulsants (mainly gabapentin). One-third of the patients also received a tricyclic antidepressant (amitriptyline).
Bokhari et al., 2012. [17]	Pilot, prospective, longitudinal survey study. Seventeen post-surgical breast surgery patients	Breast cancer (100%). Twelve patients had no non-cancer pain prior to surgery, and 5 patients had non-cancer pain prior to surgery.	Breasts	Women who developed neuropathic pain post-breast surgery (PPBS) appeared to be younger than those who did not develop PPBS (*p* = 0.029). In contrast, the independent samples t-test for BMI (*p* = 0.289) indicated no significant difference in the mean BMI of women who did and did not develop PPBS.	Canada	61.2	17 women (100%)	No treatment per se is specified for neuropathic pain.
Brzezinsk et al., 2012 [41]	Review	Not presented.	Not specified.	Not presented	Poland	47.2	-	Reduction of chemotherapy doses and follow-up of pharmacological treatment recommendations.
Casanova et al., 2015 [50]	RCT pilot study. Forty pediatric oncology patients, both sexes, between 5 and 18 years old.	Not applicable.	Not applicable.	The results of the preliminary evaluation of this pilot study of seven children undergoing treatment for 4 weeks are as follows: pain threshold of the painful limb improved by 10 points and pain perception by 3.1 points. The best results were obtained by children with leukemia (n = 2) and brain tumors (n = 1).	Spain	52.3	Does not state.	Graded motor imaging (GMI) and neural mobilization (NM).
Cofeen et al., 2019 [49]	Retrospective study: 754 patients.	233 patients with chemotherapy-induced neuropathic pain (CINP) (30.9%) and 521 with neuropathic pain due to other causes (69.1%).	Breast, blood, lung, and colon.	Cancer progression to stage 3 or 4 was more common in patients with CINP (*p* < 0.001). Paclitaxel, platinum, vincristine, and thalidomide were used more frequently in patients with CINP. The mean DN4 score was higher (*p* < 0.001), and glove and stocking pain distribution was also reported more frequently in patients with CINP (*p* < 0.001).	Mexico	36.6	525 females (69.6%) and 229 males (30.4%)	They do not show a treatment but rather determined the incidence and characteristics of chemotherapy-induced neuropathic pain.
Dou et al., 2014 [51]	Randomized double-blind clinical trial: 40 patients.	Eleven (27.5%) patients with breast cancer, 10 (25%) with lung cancer, 6 (15%) with colorectal cancer, 4 (10%) with sarcoma, 3 (7.5%) with liver cancer, and 6 (15%) with other types of cancer.	Breast, lung, colon, rectum, bone, and liver.	Based on responses to the MOS-SS, PGB treatments resulted in significantly lower scores for sleep disorders (*p* < 0.001) and sleep problems index (*p* < 0.001) and more hours of sleep (7.7 ± 1.2 h) than PL-controls ( 6.5 ± 1.4 h; *p* < 0.001).	China	39.1	24 (60%) men and 16 (40%) women	-
Expósito Vizcaíno et al., 2018 [18]	Cross-sectional observational study. Twenty-two patients with multiple myeloma	Bortezomib-induced neuropathy disability: Total no. of participants: 9 Grade 1 was 30 ± 21%, grade 2: 49 ± 18.4%, grade 3: 67 ± 21.6%, and grade 4: 0%. Pain intensity (0–10): Mean maximum pain intensity 7.8 ± 2.5. Minimum mean average pain intensity 2.4 ± 1.6. Mean average pain intensity 4.5 ± 2.3. Location: Lower extremities (toes and soles) 9 (100%). Upper extremities (hands) 5 (56%). DN-4 (nerve fiber involvement). Leeds Assessment of Neuropathic Signs and Symptoms (LANSS) pain scale: Total no. of participants: 9 10.11 ± 4.22. Neuropathy grade 1 8.33 ± 3.51. Grade 2 neuropathy 14.5 ± 6.36. Grade 3 neuropathy 9.25 ± 2.98. Grade 4 neuropathy 0%. Small fiber neuropathy (SFN) 5 (56%). Mixed peripheral neuropathy (MPN) 2 (22%). SFN = MPN 2 (22%).	White blood cells	Grade 2 was the most common (n = 5; 36%). Pain usually affected the toes and soles of the feet (100% of patients reported pain in these locations) and, to a lesser extent, the upper extremities, especially the hands (56%). No significant neuropathy was identified in 3 patients (14%) after completing treatment (grade 0 on the WHO scale). In the remaining participants (n = 8; 36%), neuropathy and/or its association with the drug could not be reliably determined.	Spain	44.2	11 (50%) males and 11 (50%) females	Bortezomib
Fallon et al., 2015 [19]	Descriptive study: 51 patients.	Thirty-one patients had chemotherapy-induced neuropathic pain.	Colon, rectum, white blood cells, lung, ovaries, breast.	31/38 (82% percent) of evaluable patients had an improvement in their pain scores. The median total BPI improved from 47 (interquartile range, 30 to 64) to 34 (6 to 59), *p* < 0.001. There were also significant improvements in the “worst pain” and pain interference subscales. All statistically significant improvements in pain were also clinically significant.	United Kingdom.	49.1	32 (61%) women and 19 (39%) men	Topical aqueous menthol cream 1% (analgesic).
Fontes et al., 2017 [20]	Prospective study 501 patients.	One hundred percent of patients with neuropathic pain. Patients undergoing chemotherapy 297 (59.3%). Stage I breast cancer 234 (46.7%). Stage II 155 (30.9%). Stage III 75 (15%). Stage IV 3 (0.6%).	Breast	Among patients with good sleep quality at baseline, there was a deterioration in sleep quality, significantly greater in those with neuropathic pain during the first year of follow-up [*p* = 0.036].	Portugal	51.5	Not specified	No treatment per se is specified.
Garzón-Rodriguez et al., 2013 [21]	Cross-sectional, non-interventional, cross-sectional, multicenter, observational study: 945 patients.	308 patients with cancer-related neuropathic pain (CRNP). The proportion of patients with CRNP in cancer patients experiencing chronic pain was thus 32.6% (95% CI 29.62 to 35.58). The percentage of patients with CRNP identified in this study was in line with the international study estimate of 39.7%. The most common cancer types in this population were breast cancer with 56 patients (18.1%) and lung cancer (14.5%) with 45 patients. 137 (44.2%) patients with CRNP had locoregional progression of cancer, and approximately half (49.0%) experienced disease metastasis to one or more sites. Bone was the most common site of metastasis.	Breast, lung, and bone.	Mean subscale scores on the m-BPI-sf for the CRNP population were 5.48 (95% CI: 5.17, 5.80, n = 252) for pain interference and 4.63 (95% CI: 4.38, 4.89, n = 256) for pain severity. Mean scores for individual items on the m-BPI-sf in the CRNP population were all between 4.08 and 6.58, with the exception of pain at least in the last 24 hours (mean = 2.92).	Spain	62.2	502 (53.12%) men and 432 (46.88%) men.	No treatment per se is presented.
Gebremedhn et al., 2019 [22]	Retrospective study: 322 patients	Patients with neuropathic pain 250 (77.6%). Cancer of the cecum 61 (18.9%). Ascending colon cancer 30 (9.3%). Transverse colon cancer 21 (6.5%). Descending colon cancer 10 (3.1%). Cancer of the sigmoid colon 109 (33.9%). Cancer of the rectum sigmoid junction 28 (8.7%). Rectal cancer 63 (19.6%).	Cecum, colon, rectum.	There was no significant difference in patient survival according to sex and cancer stage (non-metastatic and metastatic cancer). *p* = 0.145. However, middle-aged patients (51–60 years) had a relatively longer survival time compared to younger (30–50 years) and/or older (61–78 years) patient groups with a median survival of 24 months.	Australia	49.9	184 males (57.14%), 138 (42.86%) females.	They do not exhibit a treatment for neuropathic pain.
Golan-Vered and Pud, 2012 [23]	Non-randomized clinical trial: 42 patients with breast cancer who were treated with paclitaxel.	One (2.5%) patient had stage 1 cancer, 18 (45%) patients had stage 2 cancer, and 21 (52%) patients had stage 3 cancer. We identified 2 distinct groups of breast cancer patients, namely Low Cluster and High Cluster, based on their experiences with 4 highly prevalent symptoms; 50% of paclitaxel-treated patients developed CINP; a combination of CINP and clusters revealed a subgroup with no evidence of CINP within the Low Cluster group (35%) and a subgroup with CINP within the High Cluster group (22.5%). These 2 subgroups are “double lucky” and “double” unlucky”, respectively.	Breast.	Does not present.	Israel	50.6	42 (100%) women.	They do not exhibit a treatment for neuropathic pain.
Gordillo-Altamirano et al., 2016 [25]	Analytical cross-sectional study: 237 patients	58 (24.5%) with breast cancer, 40 (16.9%) with cancer of the female genitalia, 39 (16.5%) with lymphatic- hemapoytic cancer, 25 (10.5%) with digestive cancer, 75 (31.6%) with other types of cancer.	Breast, vagina, white blood cell, stomach, and other types (not specified).	Health-related quality of life (HRQOL): The physical component of HRQoL was 39.3 ± 9.1 (SD: 39.5), its subcomponents: Physical Functionality was 52.6 ± 29.2 (55), Physical Role was 39.3 ± 33.4 (31.3), Body Pain was 43.1 ± 26.6 (41), and General Health was 55.1 ± 22.7 (55).	Ecuador	57.8	69 (29.1%) men and 168 (70.9%) women.	No treatment specified.
Harada et al., 2015 [52]	Prospective observational study: 220 patients.	185 (84.1%) underwent biological treatment or chemotherapy. 18.6% of the patients had neuropathic pain.	Lung, colorectal, stomach, pancreas, lymph, bile duct, uterus, breast, liver and other types of cancer	Patients with NP were significantly younger and had significantly higher mean VAS scores than patients without NP (*p* < 0.001). There were no significant differences in sex and ECOG PE between the 2 groups of patients. There were no significant differences in survival times between patients with NP and those without NP (*p* = 0.342).	Japan	44.4	127 (57.7%) men and 93 (42.3%) women.	No treatment es displayed.
Haumman et al., 2016 [24]	Randomized clinical trial: 52 patients.	52 (100%) patients with neuropathic pain. 11 (22%) patients undergoing fentanyl chemotherapy.	Not specified.	Clinical success, defined as a 50% decrease in mean NRS, was observed in 27% of patients in the Fen group and 44% in the Met group (p Z 0.23) at 3 weeks (Figure 2). A 50% decrease in mean NRS was achieved faster with Met than with Fen. At 1 week, 50% of patients in the Met group and 15% in the Fen group showed clinical success (p Z 0.012). This initial difference between the groups after 1 week diminished over the following weeks, as no significant difference was observed between the Fen and Met groups at 3 and 5 weeks (49% Met versus 33 Fen p Z 0.367).	Holland	63.2	30 (57.69%) men and 22 (42.31%) women.	Methadone.
Jain et al., 2014 [26].	Prospective study: 300 patients of both sexes aged 18–70 years.	Approximately 10% of patients had features of acute neuropathic pain (ANP). The “painDETECT questionnaire” showed 8.3% as “possible” and 1.7% “positive” probability of prevalence of neuropathic pain in post-surgical patients during the first week of the postoperative period. There was a strong correlation between postoperative pain score and neuropathic pain score.	Chest, gastrointestinal tract, genital and urinary organs, breast, bone, soft tissues, head and neck.	A significant relationship was found between the severity of postoperative pain and the occurrence of ANP in the postoperative period (*p* < 0.05). Among the characteristics of neuropathic pain, tingling was found to be a common problem (25–60%) in different surgical subgroups.	India	39.8	Not specified.	It does not specify but analyzes neuropathic pain among the 300 patients using the “painDETECT questionnaire”.
Jiang et al., 2019 [27]	This trial is randomized, double-blind, placebo-controlled: 128 patients, with 64 in the pregabalin group and 64 in the placebo group.	Pregabalin group: Nasopharynx 28 (43.8%) patients. Oral cavity and lips 9 (14.1%) patients. Larynx 7 (10.9%) patients. Oropharynx 5 (7.8%) patients. Paranasal sinuses 2 (3.1%) patients. Other 13 (20.3%) patients. Placebo group: Nasopharynx 30 (46.9%) patients. Oral cavity and lips 8 (12.5%) patients. Larynx 10 (15.6%) patients. Oropharynx 2 (3.1%) patients. Paranasal sinuses 1 (1.6%) patients. Other 13 (20.3%) patients.	Pharynx, oral cavity, larynx.	The baseline numerical rating scale (NRS) for the pregabalin group was 6.47 (1.50); at 16 weeks, it was 4.04 (1.51), leaving a difference of −2.44 (1.52). For the placebo group, the baseline was 6.34 (1.37), and at 16 weeks, it was 4.77 (1.28), giving a difference of −1.58 (1.25). The Net Difference between groups instead (95% CI) was −0.86 (−1.35 to −0.37) with ap = 0.001. And the adjusted between-group difference in change (95% CI) was −0.87 (−1.44 to −0.30) with ap = 0.003.	China	40.0	51 (39.8%) women, 77 (60.2%) men.	Pregabalin (anticonvulsant).
Juwara et al., 2019 [28]	Prospective study: 195 patients.	Patients with post-surgical breast cancer 100%. Patients undergoing chemotherapy 141 (72.3%).	Breast	Neuropathic pain is significantly higher in patients with anxiety than in patients without anxiety *p* = 0.018. The odds of neuropathic pain are apparently higher for patients with acute pain upon moving *p* = 0.025. The baseline pain and MCL components included in the logistic model were not statistically significant. Compared to the unadjusted model, *p* = 0.035	Canada	67.1	195 (100%) women.	It does not expose a treatment to neuropathic pain.
Kajiume et al., 2012 [13]	Case report: 1 patient.	1 (100%) T-cell lymphoblastic lymphoma.	Lymph.	Does not present.	Japan	36.3	1 (100%) female	Intravenous administration of ketamine and lidocaine in combination with fentanyl.
Lee et al., 2016 [31].	Prospective exploratory study: 26 patients.	2 patients with cancer of the uterus (neuropathic pain due to bone metastasis). 3 with colon cancer (2 with peripheral neuropathy induced by chemotherapy (FOLFOX = Folinic acid + Fluorouracil + Oxaliplatin), and 1 due to bone metastasis. 2 patients with lymphoma (with chemotherapy-induced peripheral neuropathy (CHOP = Rituximab + Cyclophosphamide + Doxorubicin + Vincristine + Prednisolone). 3 patients with breast cancer (1 for pain due to bone metastasis and 2 for post-surgical pain). 4 patients with lung cancer (3 with post-surgical pain and 1 with pain due to bone metastasis). 1 patient with breast and lung cancer (postoperative pain). 2 myeloma patients with peripheral neuropathy induced by chemotherapy (Thalidomide). 1 patient with esophageal cancer with pain due to bone metastasis. 1 patient with cancer of the kidney and lung with pain due to bone metastasis. 1 patient with cancer of the rectum with pain due to bone metastasis.	Uterus, colon, breast, lung, esophagus, rectum, kidney, and white blood cells.	Calmare Therapy (CT) was effective in controlling cancer-related neuropathic pain (CNP), including chemotherapy-induced peripheral neuropathy (CIPN), metastatic bone pain, and post-surgical neuropathic pain. The decrease in NRS pain scores after two weeks of follow-up (visit 3) was statistically significant (*p*<0.001), which was obtained during the first week of TC (visit 1). The most dramatic decrease was observed after the 10th day of treatment (visit 2), with a slight increase after two weeks of follow-up (visit 3). BPI scores providing their “worst”, “minimal”, “average”, and “current” pain intensity and assessing 7 domains of overall functional status improved significantly at Visit 3. Rescue opioid consumption decreased significantly (*p* = 0.050) at Visit 3. Half of the patients (n = 10) responded that they were mildly to moderately satisfied with their CT at Visit 3.	South Korea	33.4	11 (42%) females and 15 (58%) males.	Calmare Therapy (also known as Scrambler Therapy or CT).
Levi et al., 2016 [44]	Case report.1 patient survivor of laryngeal cancer.	1 (100%) bilateral intractable pain in the distributed territory of the trigeminal mandibular branch.	Larynx	Does not present	Italy	34.9	1 (100%) male.	Stimulation of the peripheral nerve field.
Lopez-Ramirez., et al 2016 [32]	Cross-sectional observational study: 15 patients.	60% of patients with chronic neuropathic pain were unrelated to their cancer diagnosis, but all had been referred to the Radiation Therapy and Oncology Service for radiation therapy.	Breast, prostate, lung, endometrium, nasopharynx and colon.	The analgesic effect of the 5% lidocaine patch was potent in eight cases (53.33%) and partial in four cases (26.66%), which, overall, represents an analgesic efficacy rate of 79.99%.	Spain	45.5	5 (33.33%) men and 10 (66.66%) women.	The treatment for neuropathic pain is 5% lidocaine patches.
Lu, et al., 2017 [53]	Cross-sectional study: 106 participants.	86 (81.1%) colon cancer and 20 (18.9%) rectal cancer.	Colon and rectum	The correlation between the scores of the Chinese version of the CNPSI and the Chinese version of the POMS-SF questionnaires had a statistically significant positive correlation (0.082e0.429, *p* < 0.05e0.005).	China	50.1	52 (49.1%) men, 53 (50.9%) women.	-
Mañas et al., 2011 A [14]	Prospective, multicenter, epidemiological study: 296 patients.	57 (19.7%) with breast cancer, 57 (19.7%) with lung cancer, 47 (16.2%) with cancer of the genitourinary system, 44 (15.2%) with cancer of the gastrointestinal tract, 23 (7.9%) with cancer of the neck, 21 (7.2%) with cancer of the bone, 8 (2.8%) with sarcoma, 7 (2.4%) with head cancer, 7 (2.4%) with oral cavity cancer, 5 (1.7%) of unknown origin, 4 (1.4%) with multiple myeloma, 4 (1.4%) with skin cancer, 2 (0.7%) with thymus carcinoma, 2 (0.7%) with lymphoma, 1 (0.3%) with neuronal cancer, 1 (0.3%) with plasmacytoma.	Breast, lung, genitourinary system, gastrointestinal tract, neck, bones, head, oral cavity, skin, thymus, nervous system.	The most frequent location of PN was in the thoracic region (46% at baseline and 39.4% at 8 weeks; *p* = 0.0045), followed by the lumbar (32.8% at baseline and 31.9% at 8 weeks), with the sacral area being the least frequent location (19.9% and 21.3%, respectively; *p* = 0.0339)	Spain	38.8	162 (57.7%) illnesses. 134 (42.3%) women.	Non-narcotic analgesics, narcotic analgesics, benzodiazepines, antidepressants and anticonvulsants.
Mañas et al., 2011 B [48]	Randomized clinical trial: 291 patients.	108 patients were treated with chemotherapy.	Breast, lung, genitourinary system, gastrointestinal tract, neck, bone, oral cavity, head, skin, thymus, white blood cells, neurons, plasmacytes.	At 8 weeks, satisfaction with treatment was 92.6% (PGB) vs. 78.9% (non-PGB), *p* = 0.0024, and benzodiazepine use was 37.8% (non-PGB) vs. 19.8% (PGB), *p* = 0.0009.	Spain	49.9	151 (51.89%) men and 140 (48.11%) women.	Pregabalin (PGB) with combination therapy to reduce neuropathic pain in cancer patients.
Matsuoka et al., 2012 [29]	Cross- sectional descriptive study. 15 patients	6 patients with colorectal cancer (2 patients with pain due to FOLFOX (Folinic acid + Fluorouracil + Oxaliplatin), 2 due to XELOX (capecitabine + oxaliplatin), and 2 due to spinal cord invasion). 3 patients with lung cancer (2 with pain due to spinal cord invasion and 1 due to XELOX (capecitabine + oxaliplatin)). 5 patients with breast cancer (2 with pain due to PTX (paclitaxel), 1 due to PMPS (postmastectomy pain syndrome), and 1 due to spinal cord invasion).	Colon, rectum, lung, and breasts	Pain was reduced in 7 of the 15 patients, and adverse effects improved in four patients, indicating that duloxetine was effective in 11 patients. Does not present statistical data.	Japan	50.1	9 (60%) men and 6 (40%) women.	Duloxetine decreased neuropathic pain in 11 of the 15 patients.
Mulvey et al., 2017 [43]	Systematic review of the performance of screening tools and analysis of symptomatic profiles.	1351 participants. Does not specify the type of cancer	Does not specify the type of cancer	The combined prevalence of clinically diagnosed tumor-related pain was 70%, treatment-related pain was 20%, and the remaining 10% was a combination of mixed treatment- and tumor-related pain, comorbid pain, and pain of uncertain etiology.	UK	60.1	743 (55%) women and 608 (45%) men.	Does not specify treatment.
Mustonen et al., 2020 [46]	Clinical trial: 104 patients.	104 (100%) patients with breast cancer. 27 (26%) in Grade 1, 42 (40%) in Grade 2, and 35 (34%) in Grade 3.	Breast.	Comparison of results of bedside examination (BE) and quantitative sensory testing (QST). BE and corresponding QST items showed equal results in 34–57% of patients. QST z scores did not differ significantly in patients with sensory gain, sensory loss, or normal findings on the corresponding BE test. The three QST phenotypes differed very little in BE.	Finland	52.7	104 (100%) women.	It does not present a treatment but seeks to characterize static mechanical allodynia (SMA) in women with chronic post-surgical neuropathic pain (CPSNP).
Okamoto et al., 2013 [30]	Retrospective study: 46 patients.	46 (100%) patients undergoing chemotherapy.	Breasts, muscles, rectum, lung, stomach, red blood cells, ovaries, lymph nodes, liver, esophagus, uterine body, abdominal wall, and oral cavity.	The mean pain intensity decreased significantly from 7.3 + 2.0 to 3.4 + 2.3 after ketamine administration (*p* < 0.01). The number of rescue opioid administrations was significantly reduced from 7.4 + 7.2 to 3.9 + 5.9 after ketamine administration (*p* < 0.01).	Japan	56.9	25 (54.35%) men and 21 (45.65 %) women.	Gradually adjusted doses of ketamine.
Oosterling et al., 2016 [45]	Cross-sectional study: 892 patients	170 (19%) patients had neuropathic pain.	Bones, soft tissues, breast, lungs, female genital organs.	Patients receiving curative treatment (Group 2) had more frequent neuropathic symptoms (*p* = 0.004), as well as those treated with systemic drugs (*p* = 0.004), neurotoxic drugs (*p* = 0.010), radiotherapy (*p* = 0.006), or lymph node dissection (*p* = 0.013). More types of these interventions were also associated with more symptoms (*p* < 0.001).	Holland	60.1	252 (28%) men, 640 (72%) women.	It did not exhibit a DNP treatment.
Perez et al., 2014 [47]	Cross-sectional study: 358 patients.	20.4% of patients suffer from neuropathic pain. 100% underwent chemotherapy.	Not specified.	Did not present statistical values.	Spain	66.5	162 (45.25%) men and 198 (55.30%) women.	It does not present a treatment; it compares neuropathic pain detection tools (DN4 and LANSS).
Poullin et al., 2016 [34]	Cross-sectional survey: 76 patients.	76 patients who had completed taxane- or platinum-based chemotherapy for breast or gastrointestinal cancer at least 1 year before the study.	Breast and gastrointestinal tract.	Mindfulness and pain intensity together, in the first step, accounted for 36% (*p* < 0.001) of the unique variance in pain catastrophizing. In the next step, the interaction term between mindfulness and pain intensity accounted for 8% (*p* < 0.01) of the unique variance in pain catastrophizing; the association between pain intensity and pain catastrophizing decreased as the level of mindfulness increased.	Canada	54.4	58 (76.3%) women and 18 men (23.7%).	It does not indicate a treatment itself, but rather, it indicates that “mindfulness” improves the quality of life of patients with CNP (chronic neuropathic pain).
Ris-Pina et al., 2017 [37]	Cross-sectional observational study: 371 patients.	263 (71%) had metastatic disease from at least one site, and 176 (47%) had their treatment goal documented by the oncology team as palliative. Of the study sample, 30.2%, 45%, and 47.4% had received surgery, chemotherapy, and radiotherapy, respectively, within the 30-day period before their first attendance at the cancer pain clinic.	Head, neck, and lung.	Of the 371 patients, 246 (66%) were clinically evaluated as having a cancer pain syndrome primarily with mixed mechanisms; 161 (43%) scored ≥ 4 on the DN4 test. Using our study criteria definitions, we identified 120 (32.3%) of the sample with an NPC.	Portugal	53.1	199 (53.3%) women, 172 (46.7%) men.	This paper does not indicate a treatment for neuropathic pain.
Sherman et al. 2015 [35]	Cross-sectional observational study: 106 patients.	100% of breast cancer patients. 31.1% of patients underwent chemotherapy.	Breast.	There was no statistically significant difference in any of the following DASS variables (*p* = 0.95), DASS anxiety (*p* = 0.91).	Australia	60.6	106 (100%) women.	It does not present a treatment per se for neuropathic pain.
Shkodra et al., 2021 [36]	Prospective study: 350 patients.	108 patients were treated with chemotherapy. Cancer of unknown origin 43 (12%).	Breast, lung, female reproductive system, colon, rectum, pancreas, prostate, urinary tract, liver and biliary tract, head and neck.	The estimated prevalence of NcP based on Retrospective Clinical Classification was 28.6%, 95% CI (23.8–33.9%), while it was 20%, 95% CI (15.9–24.6%) according to the results of the DN4 questionnaire. Cohen’s kappa indicated moderate agreement (kappa 5 0.57, 95% CI [0.47–0.67]).	Norway	38.8	192 (54.86%) women and 158 (45.14%) men	They do not show a specific treatment but rather compare the different clinical approaches with the evaluation of neuropathic pain.
Smith et al., 2020 [38]	Randomized clinical trial: 23 patients.	23 (100%) breast cancer survivors. 13 from a group that received a therapy focused on mindfulness-based stress reduction (MBSR), and 10 from a waiting list control group.	Breast.	No significant differences in 7-day Brief Pain Inventory (BPI) short-form scores were observed between groups during any of the sessions. FiveFacet Mindfulness Questionnaire (FFMQ) change scores (post-MBSR minus pre-MBSR) were significantly higher for the MBSR group compared to controls (MBSR mean = 16.91, SD = 18.4, controls mean = 1.3, SD = 7.8, t = 2.5, *p* = 0.022).	Canada	35.5	23 (100%) women.	This study showcases a therapy focused on Mindfulness-Based Stress Reduction (MBSR), reducing pain severity non-invasively.
Sugiyama et al., 2016 [39]	Retrospective study: 28 patients.	100% of patients with neuropathic pain.	Breast, genitourinary, gastrointestinal tract, lung, head, neck, bones, soft tissues, blood and mediastinum.	-	Japan	70.1	18 (64.29%) men and 10 (35.71%) women.	This study indicates that treatment for neuropathic pain with oral methadone reduces medication consumption, relieves pain, and thus has a better quality of life.
Yanaizumi et al., 2021 [12]	Prospective observational study: 108 patients.	86 (79.6%) patients with lymph node metastasis. 45 (41.7%) patients with liver metastases. 40 (37.0%) patients with metastases in the bones. 32 (29.6%) patients with lung metastases. 27 (25.0%) patients with peritoneal metastasis.	Colon, rectum, lung, stomach, pancreas, pharynx, breasts, kidney, uterus, prostate, lymph nodes, liver, and peritoneum	Pain intensity was significantly higher in patients with PN than in patients without PN (median NRS score 7 vs. 5; *p* = 0.025).	Japan	49.9	72 (66.7%) men, 36 (33.3%) women.	This observational study does not indicate a treatment to reduce neuropathic pain.
Yesir et al., 2017 [40].	Cross-sectional observational study: 60 patients.	60 patients with breast cancer-related lymphedema (BCRL).	Breast.	There was a significant reduction in affected limb volume after treatment (*p* < 0.001). DN4, BFI, and BDI scores improved significantly after treatment (*p* < 0.001, *p* = 0.043, *p* = 0.019, respectively).	Turkey	44.8	2 (3.3%) men, 58 (96.7%) women.	Complex decongestive therapy (CDT).

**Table 2:** Association with cancer stages and treatment.

Organs	Authors	Presence of Neuropathic Pain	Receive Chemotherapy (Q), Radiotherapy (R) or PQx	Cancer Stage	Treatment for DNP
Large Intestine (Colon–Rectum)	Fallon	+	Q	N/A	+
	Open source	+	Q	N/A	−
	Lee	+	Q/PQx	IV	+
	Lopez-Ramirez	+	R	N/A	+
	Matsuoka	+	N/A	N/A	+
	Shkodra	+	Q	IV	−
	Yanaizumi	+	Q/R/PQx	IV	−
	Okamoto	+	N/A	III to IV	+
	Coffee	+	Q	I to IV (All)	−
Bone	Oosterling	+	Q/R/PQx	I to III (All)	−
	B Tricks	+	Q/R	I to IV (All)	+
	Anghelescu	+	Q/R/PQx	N/A	+
	Garzon-Rodriguez	+	Q/R	All	−
	Jain	+	PQx	N/A	−
	Sugiyama	+	N/A	N/A	+
Soft Tissue	Oosterling	+	Q/R/PQx	I to III (All)	−
	Anghelescu	+	Q/R/PQx	N/A	+
	Jain	+	PQx	N/A	−
	Sugiyama	+	N/A	N/A	+
	Okamoto	+	N/A	III to IV	+
Female Genital Organs	Oosterling	+	Q/R/PQx	I to III (All)	−
	Gordillo-Altamirano	+	N/A	I to IV (All)	−
	Shkodra	+	Q	IV	−
Moms	Oosterling	+	Q/R/PQx	I to III (All)	−
	Harada	+	Q/R/PQx	IV	−
	Tricks A	+	R	I to IV (All)	+
	Bokhari	+	PQx	0 to III (All)	−
	Fallon	+	Q	N/A	+
	Fonts	+	Q/R/PQx	0 to IV (All)	−
	Garzon-Rodriguez	+	Q/R	All	−
	Golan-Vered and Pud	+	Q	I to III (All)	−
	Gordillo-Altamirano	+	N/A	I to IV (All)	−
	Jain	+	PQx	N/A	−
	Juwara	+	PQx/Q/R	N/A	−
	Lee	+	Q/PQx	IV	+
	Lopez-Ramirez	+	R	N/A	+
	Matsuoka	+	N/A	N/A	+
	Okamoto	+	N/A	III to IV	+
	Mustonen	+	PQx	N/A	−
	Poullin	+	Q	N/A	−
	Sherman	+	PQx/Q/R	0 to III (All)	−
	Shkodra	+	Q	IV	−
	Smith	+	N/A	N/A	+
	Sugiyama	+	N/A	N/A	+
	Yanaizumi	+	Q/R/PQx	IV	−
	Yesir	+	Q/R	N/A	+
	Coffee	+	Q	I to IV (All)	−
	B tricks	+	Q/R	I to IV (All)	+
Neck	Tricks A	+	R	I to IV (All)	+
	Jain	+	PQx	N/A	−
	Jiang	+	R	N/A	+
	Shkodra	+	Q	IV	−
	King Pine	+	Q/R	IV	−
	Sugiyama	+	N/A	N/A	+
	B tricks	+	Q/R	I to IV (All)	+
Oral cavity	Tricks A	+	R	I to IV (All)	+
	Jiang	+	R	N/A	+
	Okamoto	+	N/A	III to IV	+
	B Tricks	+	Q/R	I to IV (All)	+
Lung	Oosterling	+	Q/R/PQx	I to III (All)	−
	Coffee	+	Q	I to IV	−
	Harada	+	Q/R/PQx	IV	−
	Tricks A	+	R	I to IV (All)	+
	Fallon	+	Q/R	III and IV	+
	Garzon Rodriguez	+	Q/R	All	−
	Lee	+	Q/PQx/met	IV	+
	Lopez-Ramirez	+	R	N/A	+
	B Tricks	+	Q/R	I to IV (All)	+
	Matsuoka	+	N/A	N/A	+
	Okamoto	+	N/A	III to IV	+
	Shkodra	+	Q	IV	−
	King Pine	+	Q/R	IV	−
	Sugiyama	+	N/A	N/A	+
	Yanaizumi	+	Q/R/PQx	IV	−
Liver	Harada	+	Q/R/PQx	IV	−
	Okamoto	+	Q	III to IV	+
	Shkodra	+	Q	IV	−
	Yanaizumi	+	Q/R/PQx	IV	−
Blood	Coffee	+	Q	I to lV	−
	Harada	+	Q/R/PQx	IV	−
	Kajiume	+	Q/R	III	+
	Aquazzino	+	Q/R/PQx	N/A	−
	Anghelescu	+	Q/R/PQx	N/A	+
	Exposito	+	Q	N/A	+
	Fallon	+	Q/R	III and IV	+
	Lee	+	Q/PQx/met	IV	+
	B tricks	+	Q/R	I to IV (All)	+
	Okamoto	+	Q	III to IV	+
	Sugiyama	+	N/A	N/A	+
	Gordillo-Altamirano	+	N/A	I to IV (All)	−
Stomach	Harada	+	Q/R/PQx	IV	−
	Gordillo-Altamirano	+	N/A	I to IV (All)	−
	Okamoto	+	Q	III to IV	+
	Yanaizumi	+	Q/R/PQx	IV	−
Pancreas	Harada	+	Q/R/PQx	IV	−
	Shkodra	+	Q	IV	−
	Yanaizumi	+	Q/R/PQx	IV	−
Bile duct	Harada	+	Q/R/PQx	IV	−
	Shkodra	+	Q	IV	−
Uterus	Harada	+	Q/R/PQx	IV	−
	Lee	+	Q/PQx/met	IV	+
	Okamoto	+	Q	III to IV	+
	Yanaizumi	+	Q/R/PQx	IV	−
Genitourinary	Tricks A	+	R	I to IV (All)	+
	Jain	+	PQx	N/A	−
	B Tricks	+	Q/R	I to IV (All)	+
	Sugiyama	+	N/A	N/A	+
Gastrointestinal tract	Tricks A	+	R	I to IV (All)	+
	Jain	+	PQx	N/A	−
	B Tricks	+	Q/R	I to IV (All)	+
	Poullin	+	Q	N/A	−
	Sugiyama	+	N/A	N/A	+
Head	Tricks A	+	R	I to IV (All)	+
	Jain	+	PQx	N/A	−
	B Tricks	+	Q/R	I to IV (All)	+
	Shkodra	+	Q	IV	−
	Sugiyama	+	N/A	N/A	+
	Reis-Pina	+	Q/R	IV	−
Fur	Tricks A	+	R	I to IV (All)	+
	B tricks	+	Q/R	I to IV (All)	+
Timo	Tricks A	+	R	I to IV (All)	+
	B tricks	+	Q/R	I to IV (All)	+
Nervous system	Aquazz	+	Q/R/PQx	N/A	−
	Tricks A	+	R	I to IV (All)	+
	B Tricks	+	Q/R	I to IV (All)	+
Lymph nodes	Anghelescu	+	Q/R/PQx	N/A	+
	Yanaizumi	+	Q/R/PQx	IV	−
	Okamoto	+	Q	III to IV	+
	Gordillo-Altamirano	+	N/A	I to IV (All)	−
	B Tricks	+	Q/R	I to IV (All)	+
Ovaries	Fallon	+	Q/R	III to IV	+
	Okamoto	+	Q	III to IV	+
Esophagus	Lee	+	Q/PQx/met	IV	+
	Okamoto	+	Q	III to IV	+
Prostate	Lopez-Ramirez	+	R	N/A	+
	Shkodra	+	Q	IV	−
	Yanaizumi	+	Q/R/PQx	IV	−
Larynx	Levi	+	R/PQx	N/A	+
	Jiang	+	R	N/A	+
Pharynx	Yanaizumi	+	Q/R/PQx	IV	−
	Jiang	+	R	N/A	+
	Lopez-Ramirez	+	R	N/A	+

**Table 3:** Reports of conditions and presence of neuropathic pain.

Type of Cancer, Number of Articles, and N	Neuropathic Pain Associated with Cancer	Post-Surgical Neuropathic Pain	Neuropathic Pain Associated with Chemotherapy	Difference of Means
Colorectal cancer, 9 articles (N = 1666)	2 studies (33 patients)	3 studies (32 patients)	5 studies (598 patients)	0.001
Bone cancer, 6 articles (N = 2785)	3 studies (398 patients)	4 studies (413 patients)	3 studies (64 patients)	0.45
Soft tissue cancer, 5 articles (N = 1589)	1 study (5 patients)	2 studies (336 participants)	1 study (15 participants)	0.001
Female genital cancer, 3 articles (N = 1479)	N/A	N/A	N/A	N/A
Breast cancer, 25 articles (N = 5704)	6 studies (670 patients)	11 studies (703 patients)	9 studies (359 patients)	0.32
Cervical cancer, 7 articles (N = 1759)	2 studies (408 patients)	4 studies (426 patients)	3 studies (87 patients)	0.45
Oral cavity cancer, 4 articles (N = 756)	2 studies (408 patients)	2 studies (74 patients)	2 studies (16 patients)	0.001
Lung cancer, 15 articles (N = 4937)	10 studies (2100 patients)	6 studies (232 patients)	9 studies (1877 patients)	0.21
Liver cancer, 4 articles (N = 724)	3 studies (463 patients)	1 study (58 patients)	2 studies (325 patients)	
Blood cancer, 12 articles (N = 1865)	8 studies (402 patients)	3 studies (52 patients)	4 studies (999 patients)	0.03
Stomach cancer, 4 articles (N = 611)	3 studies (280 patients)	1 studies (58 patients)	1 study (87 patients)	0.01
Pancreatic cancer, 3 articles (N = 678)	3 studies (463 patients)	1 studies (58 patients)	2 studies (325 patients)	0.10
Bile duct cancer, 2 articles (N = 570)	2 studies (391 patients)	N/A	1 study (238 patients)	0.12
Uterine cancer, 4 articles (N = 394)	3 studies (120 patients)	2 studies (65 patients)	1 studies (87 patients)	0.09
Genitourinary cancer, 4 articles (N = 1701)	N/A	1 studies 30 patients	N/A	N/A
Gastrointestinal tract cancer, 5 articles (N = 1746)	N/A	1 studies (30 patients)	1 studies (291 patients)	0.001
Head cancer, 6 articles (N = 2171)	1 studies (350 patients)	2 studies (87 patients)	3 studies (559 patients)	0.06
Skin cancer, 2 articles (N = 1371)	N/A	N/A	1 studies (291 patients)	N/A
Thymus cancer, 2 articles (N = 1371)	N/A	N/A	1 studies (291 patients)	N/A
Nervous system cancer, 3 articles (N = 1509)	1 studies (78 patients)	N/A	2 studies (415 patients)	0.001
Lymph node cancer, 5 articles (N = 740)	3 studies (306 patients)	2 studies (84 patients)	3 studies (306 patients)	0.52
Ovarian cancer, 2 articles (N = 97)	1 studies (41 patients)	1 studies (19 patients)	1 studies (31 patients)	0.43
Esophageal cancer, 2 articles (N = 66)	1 studies (7 patients)	1 studies (7 patients)	N/A	N/A
Prostate cancer, 3 articles (N = 473)	3 studies (423 patients)	2 studies (60 patients)	3 studies (347 patients)	N/A
Laryngeal cancer, 2 articles (N = 175)	1 studies (64 patients)	2 studies (45 patients)	1 studies (97 patients)	N/A
Pharyngeal cancer, 3 articles (N = 297)	3 studies (137 patients)	3 studies (104 patients)	3 studies (137 patients)	0.05

## Supplementary Materials

The following supporting information can be downloaded at www.mdpi.com/article/10.3390/diagnostics15010116/s1. Table S1: Details of the search strategy.
